# Notes and News

**Published:** 1894-03-03

**Authors:** 


### Notes and News.

Collected and Communicated.

### MEETINGS OF THE WEEK.

Philanthropic Society, Redhill: Annual General Court, Cannon Street Hotel, March 2nd.

Female Orphan Asylum, Beddington: Special General Meeting, 32, Essex Street, W.C., Friday, March 2nd, 3 p.m.

Homes for Little Boys: Festival Dinner, Hotel Metropole, Saturday, March 3rd.

Samaritan Free Hospital, Marylebone Road: Annual General Meeting, March 6th, at 4.30 p.m.

National Hospital Diseases of the Heart and Paralysis, 32, Soho Square: Annual General Meeting, March 7th, 3.30 p.m.

The study of Bacteriology is spreading rapidly. The Hungarian Government have established an institute at Buda-Pest for the pursuance of bacteriological researches, especially in relation to infectious diseases. It is to benefit both public institutions and private investigators.

At a recent meeting of the managers of the Royal Edinburgh Infirmary, Dr. Muirhead moved for an obviously necessary supervision by the medical managers of the duration of the residence of patients within the hospital. Although it is obvious that to curtail a stay is a short-sighted policy in the interests of the patients and the infirmary, yet too lax procedure in the matter undoubtedly limits the benefits an institution can confer. The average duration of the stay of patients in different hospitals differs much, also it may vary in the case of one particular institution from year to year; but, as in the present case at Edinburgh, where the average visit during 1893 equalled 31½ days, some closer supervision certainly seems necessary. At Glasgow, we notice at one institution the average was as low as 16·36, a vast discrepancy, whilst at the Essex and Colchester Hospital an average of 39 was reached. It is obvious and well that at such institutions as have no convalescent branch to assist their work the duration of many cases is increased.

Professor August Weismann's work on Heredity has excited so much attention that his delivery of the Romanes Lecture, at Oxford, on May 2nd, will be hailed with a great amount of interest.

Mr. Passmore Edwards laid the foundation-stone of a new wing of the West Ham General Hospital, of which he is with great generosity defraying the cost. The vicinity is populous and poor, and the surgical work at the hospital is very heavy. The further accommodation is much needed, and is expected it will be provided at a cost of £3,000. A larger income will be necessary when the extension is made. At the festival dinner following on the laying of the foundation-stone £770 were subscribed.

The Commissioners of the borough of Leith have met with an attack on account of their Small-pox Hospital, which it is surprising that other authorities do not more frequently encounter in like cases. A patient was removed to the hospital at the instigation of the medical officer of the local authorities, but with a reluctant, if any, consent on her part. She, at the termination of her illness, claimed damages for forcible removal, and for the cost of her removal again to her home. The case, being based largely on statement and denial, is not yet decided.

A new wing in connection with the Belfast Hospital for Consumption was opened on the 16th ult. This addition is made by acquiring a house adjoining to the hospital, and by so doing the number of beds in the hospital is doubled. Unfortunately, at Belfast the need of hospital accommodation for consumptives is very large, and although an entirely new hospital at Ballynafeigh is projected, it was found necessary to extend the premises of the present hospital. The employés of several factories are subscribing definite sums to secure the privilege of prior admission.

An American medical journal has raised the question as to the benefit to doctors themselves of the custom of rendering medical aid gratuitously amongst the profession. This journal wholly advocates the continuance of the system; whilst another journal, commenting on the matter, also advises a doctor when ill to pay if he can, should this illness call for a serious amount of work and attention. Doctors have proverbially little faith in each others drugs, and it is not impossible that were the advice given them regarded in a strictly businesslike manner, more attention might be paid to it.

An adjourned quarterly court was held last week at the Addenbrooke's Hospital, Cambridge, at which the new rules were under consideration. Briefly, they relate to the appointment of physician and assistant-physicians, the powers of the dispenser, rules for the new office of assistant-matron, the addition of clauses relating to the privileges of contributing societies and individuals, and the exclusion of incurable cases. The new rules relate also to the nursing department, and these are enumerated in our "Nursing Supplement." During the discussion that followed the reading of the proposed alterations and additions in the regulations several governors objected to passing rules without due consideration, many of them having been seen for the first time on the morning of the meeting. The final decision is, therefore, adjourned to the next quarterly court.

